# Comparison of Electrospray Ionization and Atmospheric Chemical Ionization Coupled with the Liquid Chromatography-Tandem Mass Spectrometry for the Analysis of Cholesteryl Esters

**DOI:** 10.1155/2015/650927

**Published:** 2015-03-19

**Authors:** Hae-Rim Lee, Sunil Kochhar, Soon-Mi Shim

**Affiliations:** ^1^Department of Food Science and Technology, Sejong University, 98 Gunja-dong, Seoul 143-747, Republic of Korea; ^2^Department of Lipids & Off-Flavors, Nestlé Research Center, Nestec, Vers-chez-les-Blanc, 1000 Lausanne 26, Switzerland

## Abstract

The approach of two different ionization techniques including electrospray ionization (ESI) and atmospheric pressure chemical ionization (APCI) coupled with liquid chromatography-tandem mass spectrometry (LC-MS/MS) was tested for the analysis of cholesteryl esters (CEs). The retention time (RT), signal intensity, protonated ion, and product ion of CEs were compared between ESI and APCI. RT of CEs from both ionizations decreased with increasing double bonds, while it increased with longer carbon chain length. The ESI process generated strong signal intensity of precursor ions corresponding to [M+Na]^+^ and [M+NH_4_]^+^ regardless of the number of carbon chains and double bonds in CEs. On the other hand, the APCI process produced a protonated ion of CEs [M+H]^+^ with a weak signal intensity, and it is selectively sensitive to detect precursor ions of CEs with unsaturated fatty acids. The ESI technique proved to be effective in ionizing more kinds of CEs than the APCI technique.

## 1. Introduction

Mother's milk is widely known as the first and essential food for infants [1]. Many studies have discovered that mother's milk provides numerous beneficial health effects including improving neurologic development, immune system against pathogens, gastrointestinal function, and obesity inhibition [1–4]. Mother's milk consists of various nutrients including cholesterol (Chl), and such components are dependent on the mother's diets and are required for infant's growth [5]. A previous study suggested that a high level of Chl intake during infancy through mother's milk can reduce the blood Chl level in adults, implying a high amount of Chl intake can decrease the risk of atherosclerosis and heart disease [5, 6].

**Figure 1:**
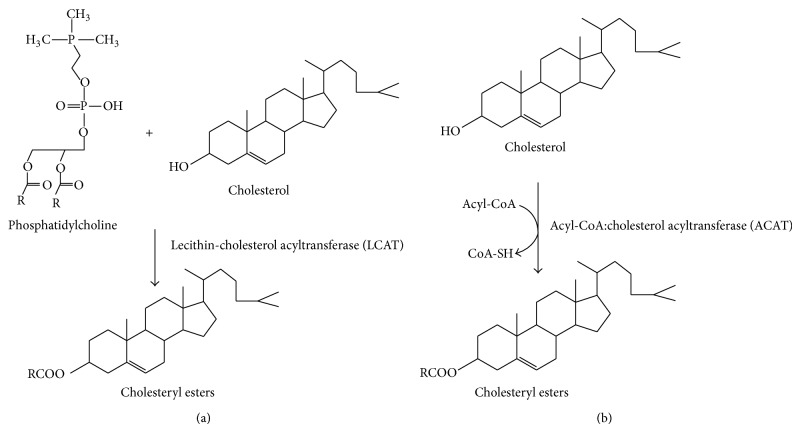
Biosynthesis of cholesterol esters (CEs) in human by two enzymes: lecithin-cholesterol acyl transferase (LCAT) (a) and acyl-coA:cholesterol acyltransferase (ACAT) (b).

Cholesteryl ester (CEs) is an esterified form of Chl in mother's milk and it consists of a long chain fatty acids, connecting with the hydroxyl group of Chl. It is known as an efficient form to transport Chl through the blood stream [7]. There are two enzymes involved in the biosynthesis of CEs in humans, that is, lecithin-cholesterol acyl transferase (LCAT) and acyl-coA:cholesterol acyltransferase (ACAT). LCAT catalyzes Chl to cholesteryl esters by transferring fatty acids to Chl. In the small intestine, absorbed Chl is esterified by ACAT (Figure 1) [7–10]. The biosynthesis of CEs plays a role in the regulation of cholesterol transport and storage as well as membrane function. More importantly, it is controlled by intracellular Chl levels [11].

Many approaches have been developed that are well-suited for analyzing hydrophobic components. Gas liquid chromatography (GC) and thin-layer chromatography (TLC) have been utilized for the analysis of CEs in human milk [7, 12, 13]. Recently, high performance liquid chromatograph (HPLC) condition has been optimized for the identification and quantification of CEs in various matrices such as human meibum, human plasma, and margarine spread [14–16]. For instance, a hexyl-phenyl HPLC column with a mobile phase consisting mixture of acetonitrile and water was used with an atmospheric pressure chemical ionization (APCI) source to analyze the CEs in food matrices such as orange juice and margarine spread [13]. Butovich [15] utilized a reversed-phase (RP) C18 HPLC column with a mobile phase mixture coupled ammonium formate, acetonitrile, and propan-2-ol with an APCI source for the identification of 20 kinds of CEs in human meibum. To date, electrospray ionization (ESI) and APCI are the most common ionization sources for the coupling of LC to a tandem mass spectrometry (MS/MS) [16]. Under optimal ESI conditions, a charged liquid is formatted and sprayed for evaporating the solvent. And then, ion formation occurs in the fission of charged droplets due to the high field intensity. This technique is appropriate for analyzing polar components [17]. In optimized APCI, a mixture of solvent molecules and analyte molecules goes through a corona discharge after being dried in the gas phase. The solvent molecules are ionized to create charged solvent ions. The charge which is located with solvent ions is transferred to the analyte molecules, producing analyte ions. APCI is usually used to analyze nonpolar molecules with lower molecular weight [18]. Hence, we hypothesized that the ESI process is more adequate ionization for analysis of CEs than the APCI due to the ESI and APCI different mechanism of ionization, a potential polarity of CEs attributed to the ester group, and CEs' large molecular weight.

## 2. Materials & Method

### 2.1. Chemicals

HPLC grade acetonitrile, propan-2-ol, methanol, and water were purchased from Fisher scientific (Leicestershire, UK). Chloroform, n-hexane, ethanol, ammonium acetate, petroleum benzene, and diethyl ether were obtained from Merck-chemicals (Darmstadt, Germany). Ammonium formate and acetic acid glacial were obtained from Biosolve (Dieuze, France).

Twenty three standards of cholesterol esters (CEs) including; Chl-butyrate, Chl-valerate, Chl-heptanoate, Chl-caprylate, Chl-nonanoate, Chl-caprate, Chl-undecanoate, Chl-laurate, Chl-tridecanoate, Chl-myristate, Chl-pentadecanoate, Chl-palmitate, Chl,-heptadecanoate, Chl-nonadecanoate, Chl-arachidate, Chl-heneicosanoate, Chl-behenate, and Chl-lignocerate were purchased from Nu-Chek (Elysian, MN). Chl-arachidonate, Chl-linoleate, Chl-palmitelaidate, Chl-oleate, and Chl-stearate were purchased from Sigma Aldrich (St. Louis, CA).

### 2.2. Preparation of Standard Solution

Aliquot amount of each standard was weighed and solubilized in 100% chloroform. Stock solution was consequently diluted by n-hexane/propan-2-ol (1 : 1, v/v) for calibration by HPLC-MS/MS (ThermoFisher Scientific, Franklin, MA).

### 2.3. Analytical Conditions

#### 2.3.1. High-Performance Liquid Chromatography (HPLC)

The method from Butovich [15] was adopted with certain modifications. The samples were analyzed by using HPLC (ThermoFisher Scientific, Franklin, MA) with aria OS software (ThermoFisher Scientific, Franklin, MA). Hypersil Gold C18 column (150 mm × 2.1 mm, 5 *μ*m) obtained from Thermo Electron (San Jose, CA) was used for the separation of CEs. Acetonitrile containing 5% of 5 mM aqueous ammonium formate was used as mobile phase A, whereas propan-2-ol contains 5% of 5 mM ammonium formate as mobile phase B. Before the injection, the column was preequilibrated with a solvent mixture (A : B, 47.4 : 52.6, v/v). The gradient rate was linearly changed to 7.6% of mobile phase A over the period of 35 min. The gradient rate was maintained for 10 min and then went back to the initial condition having 47.4% of mobile phase A within the next 1 min. It was reequilibrated for another 14 min.

#### 2.3.2. Mass Spectrometry (MS) Condition

MS was conducted after separation by HPLC using Thermo LTQ having interchangeable ESI and APIC probes (Thermo Fisher Scientific Inc., San Jose, CA). The full scan with speed in events per second was carried out.

*(1) Atmospheric Pressure Chemical Ionization Source (APCI).* The entire flow was directed to the APCI ion source operating in the positive ion mode. Total ion chromatograms were recorded in the* m/z* range of 50 to 800. The vaporization and capillary temperature were set at 270 and 250°C, respectively. Sheath, ion sweep, and auxiliary gas pressure were set at 20, 2.0, and 5 psi, respectively. In MS_2_ (MS/MS) experiments, the normalized collision energy was optimized for each of the compounds. Helium was used as a collision gas.

*(2) Electrospray Ionization Source (ESI).* The entire flow was directed to the Thermo LTQ ESI ion source operating in the positive ion mode (Thermo Fisher Scientific Inc., San Jose, CA). Total ion chromatograms were recorded in the* m/z* range of 50 to 800. ESI probe ion was used. Spray voltage was set to 4000 V. Vaporization and capillary temperature was set at 240 and 280°C, respectively. Sheath (N_2_), ion sweep, and auxiliary gas (N_2_) pressure were, respectively, set at 10, 2.0, and 5 psi. In MS2 (MS/MS) experiments, the normalized collision energy was optimized for each of the compounds. The particular transitions, the collision energy, and the tube lens settings were specific for each analyte and obtained using the TSQ Tune Master software in the optimization MS + MS/MS mode. These were shown in Table 1.

## 3. Results and Discussion

### 3.1. Comparison of Retention Time, Intensity, and Ion Fragmentation of CEs between ESI and APCI Mass Spectra

ESI is one of the primary ionization techniques for the coupling of LC to MS, while APCI is a supplementary technique to electrospray and suitable for thermally stale polar and nonpolar compounds due to no generation of charged ions. ESI is particularly suited for polar organic compounds and is sensitive to matrix effects. High molecular weight compounds can be observed as multicharged molecular ions in ESI. In contrast to ESI, the APCI technique is used to analyze smaller molecular compared to ESI technique [16, 19, 20]. Owing to different ionization mechanism and characteristics of CEs, we hypothesized that ESI is more suitable for isolation and identification of CEs than APCI; firstly, polarity of CEs is due to the ester group. Secondly, the least molecular weight of CEs is 428.7, which is combined cholesterol with acetic acid (C2:0).

The retention time (RT) of CEs by using both ESI and APCI process is expressed in Table 1. Overall, RT of CEs on total ion chromatogram (TIC) was affected by a number of carbon chains and double bonds. The Chl-lignocerate (C24:0) appeared at 28.49 min of RT, while Chl-myristate was separated at 16.06 min of RT. RT increased with longer carbon chain lengths. CEs containing the same number of carbons with different number of double bonds such as Chl-linoleate (C18:2), Chl-oleate (C18:1), and Chl-stearate (C18:0) appeared at 15.55, 18.13, and 21.31 min of RT, respectively. The present double bonds reduced RT. These findings are similar to a previous study in which fatty acid's chain length and double bond influenced the RT in the analysis fatty acid [21, 22].

Under ESI technique, both full and product ion scan of CEs standards were demonstrated to generate protonated molecular ions such as [M+Na]^+^ and [M+NH_4_]^+^ (Table 1). Among the protonated ions, [M+Na]^+^ was the most abundant for 18 CEs:* m/z* 479 for Chl-butyrate,* m/z* 493 for Chl-valerate,* m/z* 521 for Chl-heptanoate,* m/z* 535 for Chl-caprylate,* m/z* 549 for Chl-nonanoate,* m/z* 563 for Chl-caprate,* m/z* 577 for Chl-undecanoate,* m/z* 519 for Chl-laurate,* m/z* 605 for Chl-tridecanoate,* m/z* 619 for Chl-myristate,* m/z* 633 for Chl-pentadecanoate,* m/z* 647 for Chl-palmitate,* m/z* 661 for Chl-heptadecanoate,* m/z* 675 for Chl-stearate,* m/z* 689 for Chl-nonadecanoate,* m/z* 703 for Chl-arachidate,* m/z* 717 for Chl-heneicosanoate, and* m/z* 731 for Chl-behenate. As shown in Table 1, 5 other CEs including Chl-palmitelaidate, Chl-linoleate, Chl-oleate, Chl-arachidonate, and Chl-lignocerate were detected corresponding to [M+NH_4_]^+^ at* m/z* 690, 666, 640, 668, and 754, respectively. This adduct ion ([M+NH_4_]^+^) appeared to have stronger intensity than other proton adducts such as [M+Na]^+^ and [M+H]^+^. In the HPLC-ESI-MS/MS analysis of CEs, all standards lost its fatty acid as well as created a specific fragment with* m/z* 369 derived from Chl. The product ion at* m/z* 369 is supposed to be Chl upon its dehydration, corresponding to [M-H_2_O+H]^+^ and suggesting that Chl produces the specific daughter ion with an* m/z* 369 by using ESI technique. Up to now, only 20 kinds of CEs in human medium have been analyzed by APCI linked to HPLC-MS [15]. To our knowledge, there is no study regarding the analysis of various CEs using ESI as a source.

APCI ion source hardware is quite similar to that of ESI. The differences are both the APCI probe, which consists of a heated ceramic tube where the effluent is evaporated and a corona needle [23]. Thus, the difference in signal intensity and product ion of CEs between APCI and ESI source was investigated. In the current study, 23 CEs were analyzed according to the APCI technique (Table 1). All CEs standards created the protonated molecule, [M+H]^+^, showing a lower signal intensity at the same concentration compared to the consequence of the ESI source. This implies that the insufficient parent ion of CEs was produced using APCI. Nevertheless, CEs containing double bonds were observed to have relatively strong signal intensity from APCI source. For example, signal intensity of Chl-arachidonate (C20:4) was higher than that of Chl-arachidate (C20:0) in the MS_1_ experiment of their [M+H]^+^ ions. The same patterns occurred among Chl-linoleate (C18:2), Chl-oleate (C18:1), and Chl-stearate (C18:0) as shown in Table 1. The precursor ion of CEs with unsaturated fatty acids seems to be sensitively detected compared to CEs with saturated fatty acid by the APCI technique. Butovich [15] found that the fragmentation of CEs containing saturated fatty acids did not generate clear specific product ions except for* m/z* 369 because of the very low intensity of their precursor ions [M+H]^+^.

In conclusion, the ESI technique produced two protonated ions of CEs such as [M+Na]^+^ and [M+NH_4_]^+^ with strong signal intensity; otherwise, the APCI technique generated protonated ion [M+H]^+^. The ESI process coupled with LC-MS more effectively ionized CEs than the APCI process regardless of number of carbon chains and double bonds. However, there is a limitation for comparison in the ESI and APCI source regarding which one is appropriate ionization on analyzing of CEs. It is necessary to study the comparison in the limit of detection, limit of quantification, matrix effects of ESI, and APCI for providing a suitable MS to LC conditions for CEs.

## Figures and Tables

**Table 1:** Time-scheduled SIM conditions, *m*/*z* ions and corresponding structures used in the LC-ESI-MS/MS analysis of CEs.

Compound	Retention time (RT, min)	Molecular weight (Mw)	SRM transitions (*m*/*z*) Precursor ion → Product ion		Coll. energy (v)		Corresponding structure		NL		Fatty acid
			ESI	APCI	ESI	APCI	ESI	APCI	ESI	APCI	
Chl-butyrate	7.02	456.8	479.4 → 119	457.5 → 105.20	40	62	[M+Na]^+^	[M+H]^+^	2.82*E* + 03	1.22*E* + 02	4:0
			479.4 → 369	457.5 → 369	5	7					

Chl-valerate	7.69	470.8	493.4 → 105	471 → 369	39	20	[M+Na]^+^	[M+H]^+^	8.14*E* + 03	9.54*E* + 01	5:0
			493.4 → 369	—	9						

Chl-heptanoate	8.93	498.8	521.4 → 147	499.6 → 369	31	8	[M+Na]^+^	[M+H]^+^	1.28*E* + 04	1.14*E* + 01	7:0
			521.4 → 369	—	9						

Chl-caprylate	9.77	512.8	535.5 → 147	514 → 369	22	10	[M+Na]^+^	[M+H]^+^	1.44*E* + 04	2.94*E* + 01	8:0
			535.5 → 369	—	13						

Chl-nonanoate	10.64	526.9	549.5 → 105	528 → 369	42	5	[M+Na]^+^	[M+H]^+^	1.24*E* + 04	6.03*E* + 01	9:0
			549.5 → 369	—	10						

Chl-caprate	11.64	540.9	563.5 → 107	542 → 369	30	11	[M+Na]^+^	[M+H]^+^	1.50*E* + 04	2.88*E* + 02	10:0
			563.5 → 369	—	18						

Chl-undecanoate	12.51	555	577.5 → 161	556 → 369	32	8	[M+Na]^+^	[M+H]^+^	1.76*E* + 04	3.42*E* + 02	11:0
			577.5 → 369	—	14						

Chl-laurate	13.68	569	591.5 → 161	570 → 146.9	32	32	[M+Na]^+^	[M+H]^+^	1.50*E* + 04	1.40*E* + 03	12:0
			591.5 → 369	570 → 369	13	10					

Chl-arachidonate	13.98	673.11	690.6 → 109	674 → 147.14	43	36	[M+NH_4_]^+^	[M+H]^+^	5.03*E* + 05	2.46*E* + 04	20:4
			690.6 → 369	674 → 369	10	14					

Chl-tridecanoate	15.09	583	605.5 → 105	584 → 369	46	10	[M+Na]^+^	[M+H]^+^	1.22*E* + 04	1.96*E* + 02	13:0
			605.5 → 369	—	13						

Chl-linoleate	15.55	649.08	666.6 → 161	650 → 373.26	30	15	[M+NH_4_]^+^	[M+H]^+^	2.77*E* + 05	1.14*E* + 04	18:2
			666.6 → 369	650 → 361.26	9	15					

Chl-palmitelaidate	15.92	623.05	640.6 → 108	624 → 335.21	35	15	[M+NH_4_]^+^	[M+H]^+^	6.73*E* + 04	4.10*E* + 03	16:2
			640.6 → 369	624 → 369	9	13					

Chl-myristate	16.06	597	619.5 → 108	598 → 369	39	14	[M+Na]^+^	[M+H]^+^	1.57*E* + 04	2.61*E* + 02	14:0
			619.5 → 369	—	16						

Chl-pentadecanoate	17.52	611.1	633.6 → 369	612 → 369	12	5	[M+Na]^+^	[M+H]^+^	5.06*E* + 03	1.75*E* + 03	15:0

Chl-oleate	18.13	651.1	668.6 → 147	652 → 363.28	33	20	[M+NH_4_]^+^	[M+H]^+^	1.62*E* + 05	8.79*E* + 03	18:1
			668.6 → 369	652 → 369	8	11					

Chl-palmitate	18.6	625.1	647.6 → 147	626 → 161.1	28	23	[M+Na]^+^	[M+H]^+^	1.17*E* + 04	2.23*E* + 03	16:0
			647.6 → 369	626 → 369	14	11					

Chl-heptadecanoate	19.92	639.1	661.6 → 105	640 → 146.89	69	32	[M+Na]^+^	[M+H]^+^	1.43*E* + 04	1.67*E* + 03	17:0
			661.6 → 369	640 → 369	16	10					

Chl-stearate	21.31	653.1	675.6 → 135	654 → 273.16	38	31	[M+Na]^+^	[M+H]^+^	9.78*E* + 03	7.03*E* + 02	18:0
			675.6 → 369	654 → 369	15	6					

Chl-nonadecanoate	22.53	667.2	689.6 → 119	668 → 147	45	28	[M+Na]^+^	[M+H]^+^	1.24*E* + 04	7.34	19:0
			689.6 → 369	668 → 369	19	9					

Chl-arachidate	23.85	681.2	703.6 → 335	682 → 228.75	16	33	[M+Na]^+^	[M+H]^+^	7.32*E* + 04	6.00*E* + 02	20:0
			703.6 → 369	682 → 369	15	5					

Chl-heneicosanoate	25.14	695.2	717.7 → 349.5	696 → 272.6	17	31	[M+Na]^+^	[M+H]^+^	5.94*E* + 04	2.92*E* + 03	21:0
			717.7 → 369	696 → 369	19	14					

Chl-behenate	26.39	709.2	731.7 → 363	710 → 386.13	17	14	[M+Na]^+^	[M+H]^+^	6.73*E* + 04	1.82*E* + 03	22:0
			731.7 → 369	710 → 369	23	9					

Chl-lignocerate	28.49	737.3	754.7 → 132	738 → 108.96	50	28	[M+NH_4_]^+^	[M+H]^+^	4.86*E* + 04	5.01*E* + 03	24:0
			754.7 → 369	738 → 369	11	16					
